# Long-Term Supranutritional Supplementation with Selenate Decreases Hyperglycemia and Promotes Fatty Liver Degeneration by Inducing Hyperinsulinemia in Diabetic *db/db* Mice

**DOI:** 10.1371/journal.pone.0101315

**Published:** 2014-07-01

**Authors:** Chaoqun Wang, Shulin Yang, Ningbo Zhang, Yulian Mu, Hongyan Ren, Yefu Wang, Kui Li

**Affiliations:** 1 College of Life Sciences, Wuhan University, Wuhan, Hubei, P. R. China; 2 State Key Laboratory for Animal Nutrition, Institute of Animal Science, Chinese Academy of Agricultural Sciences, Beijing, P. R. China; Institute of Medical Research A Lanari-IDIM, University of Buenos Aires-National Council of Scientific and Technological Research (CONICET), Argentina

## Abstract

There are conflicting reports on the link between the micronutrient selenium and the prevalence of diabetes. To investigate the possibility that selenium acts as a “double-edged sword” in diabetes, cDNA microarray profiling and two-dimensional differential gel electrophoresis coupled with mass spectrometry were used to determine changes in mRNA and protein expression in pancreatic and liver tissues of diabetic *db/db* mice in response to dietary selenate supplementation. Fasting blood glucose levels increased continuously in *db/db* mice administered placebo (DMCtrl), but decreased gradually in selenate-supplemented *db/db* mice (DMSe) and approached normal levels after termination of the experiment. Pancreatic islet size was increased in DMSe mice compared with DMCtrl mice, resulting in a clear increase in insulin production and a doubling of plasma insulin concentration. Genes that encode proteins involved in key pancreatic β-cell functions, including regulation of β-cell proliferation and differentiation and insulin synthesis, were found to be specifically upregulated in DMSe mice. In contrast, apoptosis-associated genes were downregulated, indicating that islet function was protected by selenate treatment. Conversely, liver fat accumulation increased in DMSe mice together with significant upregulation of lipogenic and inflammatory genes. Genes related to detoxification were downregulated and antioxidant enzymatic activity was reduced, indicating an unexpected reduction in antioxidant defense capacity and exacerbation of fatty liver degeneration. Moreover, proteomic analysis of the liver showed differential expression of proteins involved in glucolipid metabolism and the endoplasmic reticulum assembly pathway. Taken together, these results suggest that dietary selenate supplementation in *db/db* mice decreased hyperglycemia by increasing insulin production and secretion; however, long-term hyperinsulinemia eventually led to reduced antioxidant defense capacity, which exacerbated fatty liver degeneration.

## Introduction

Selenium is a necessary trace element in the body that is currently used as a nutritional supplement for humans and animals, although whether it is beneficial has remained a subject of controversy. Initially considered a toxin, during the mid-20th century researchers discovered that selenium exerts positive effects on human and animal health and can thus be either beneficial or detrimental [1], [2], acting as a “double-edged sword”. Further studies have demonstrated that the safe range of selenium intake is very narrow and that the effects of selenium on health follow a U-shaped risk curve [2], [3]. While the negative health consequences [3], [4] of dietary selenium deficiency (e.g., Keshan disease and Kashin-Beck disease) and selenium excess (e.g., hair loss, brittle, thickened and stratified nails) have been noted, the effects of intermediate levels of selenium are less certain.

There are conflicting reports on the link between selenium micronutrient status and the prevalence of type 2 diabetes [5]. On the one hand, selenium can act as an antioxidant nutrient in different cell types via incorporation of selenocysteine into selenoproteins through a complex genetic mechanism encoded by the UGA codon [6], and thereby contribute to the prevention of cardiovascular disease, cancer, and diabetes [7], [8]. Furthermore, selenium has insulin-like properties and could been qualified as a potential antidiabetic agent [9]. Many studies have demonstrated a protective effect of selenium against type 1 and type 2 diabetes [8], and the use of appropriate selenium supplements may improve glucose metabolism by alleviating hyperglycemia, regulating glycolysis and gluconeogenesis, and activating key components of the insulin signaling cascade [10], [11]. On the other hand, more recent findings from large-scale human studies [12]–[14] and animal experiments [15]–[17] have shown that high selenium status or intake is positively correlated with an increased risk of type 2 diabetes. Thus, it may be important to examine the effect of selenium supplementation on the development of type 2 diabetes. In patients with type 2 diabetes, selenium causes adverse effects on blood glucose homeostasis, even when the plasma selenium concentration is raised from “deficient” levels to the optimal concentration for antioxidant activity [18]. Thus, regarding the pathologies involved in type 2 diabetes, selenium may act as a “double-edged sword”, and therefore the detailed molecular mechanism that underlie how selenium promotes or prevents the development of type 2 diabetes require further investigation.

Sodium selenite and sodium selenate are the most commonly used inorganic selenium compounds for dietary selenium supplementation. Sodium selenate is a more effective insulin mimetic than either sodium selenite or organoselenium compounds such as selenomethionine [19]–[21]. In the present study, we designed experiments to determine whether selenium acts as a “double-edged sword” in type 2 diabetes. For this purpose, we administered daily oral sodium selenate at a moderate dose for 9 weeks to *db/db* mice, which are a model system for the development of spontaneous type 2 diabetes. Genetic microarray and proteomic analyses were used to determine the effects of selenate on transcription and translation, as well as to decipher the possible mechanisms underlying the effects of long-term supranutritional selenate supplementation.

## Methods and Materials

### Animals and sodium selenate supplementation

Seven-week-old male C57BL/KsJ-*lepr^db^/lepr^db^* diabetic (*db/db*) mice were purchased from the Model Animal Research Center of Nanjing University (Nanjing, China) and were housed in a standard specific-pathogen free animal feeding room and provided free access to food and water. After habituation, mice were randomly assigned to two groups as follows: the control group (DMCtrl, *n* = 8) and the selenate supplementation group (DMSe, *n* = 8). All the animals were fed a standard mouse chow which met the basic nutritional requirements for mice, while diet made with sodium selenite contained 0.2 mg selenium per kilogram. Sodium selenate was purchased from Sigma-Aldrich (Shanghai, China) and dissolved in sterile water. The mice in the DMSe group were administered 0.8 mg sodium selenate per kilogram body weight (BW) via daily tube feeding, and the control mice were given an equal volume of sterile water. BWs were measured once per week to allow for dose adjustment. Blood was obtained by tail incision and used to determine glucose concentrations using a glucometer (OneTouch Ultra, LifeScan, Milpitas, California) once every 2 weeks after an overnight fast.

After 9 weeks, the mice in all experimental groups were anaesthetized by intraperitoneal injection of sodium pentobarbital (Sigma-Aldrich, Shanghai, China) at a dose of 50 mg/kg BW and subsequently decapitated. Blood samples were collected and pancreatic and liver tissues were immediately removed, weighed, rinsed with cold physiological saline, frozen in liquid nitrogen, and stored at −80 °C. Small pieces of these tissues were fixed in 4% paraformaldehyde for histopathological studies. Liver samples used for hepatic glycogen analysis were fixed in 85% ethanol.

The Institutional Animal Care and Use Committee at Wuhan University approved this study, which was conducted in accordance with the guidelines of the National Institutes of Health (Bethesda, Maryland, USA) for animal care.

### Biochemical assays

Insulin levels in plasma and pancreatic tissue homogenates were determined using an Enzyme Linked Immunosorbent Assay (ELISA) kit (Nanjing Jiancheng Bioengineering Institute, Nanjing, China). Analysis of glycosylated hemoglobin (HbA1c) content was performed on 100 µl of fresh total blood using a turbidimetric immunoassay performed with a Hitachi 7080 (Tokyo, Japan) automatic biochemistry analyzer. Plasma triglyceride (TG), total cholesterol (TC), and low-density lipoprotein (HDL) levels were measured using the Hitachi 7080 system with the cognate kits.

Kits purchased from Nanjing Jiancheng Bioengineering Institute were used to measure malondialdehyde (MDA) levels, catalase (CAT) activity, superoxide dismutase (SOD) activity, glutathione peroxidase (GSH-Px) activity, and reduced glutathione (GSH) levels in plasma or liver tissue homogenates. An anthrone-sulfuric acid colorimetric method [22] was used to determine liver glycogen content.

### Histopathological analysis

Liver and pancreatic tissues were dehydrated, embedded in paraffin, and cut into 6-µm thick sections. Liver samples were subjected to conventional hematoxylin and eosin (H&E) staining and periodic acid-Schiff (PAS) staining for glycogen, and pancreatic samples were stained using the conventional H&E method. Immunohistochemical analysis of pancreatic islets was performed using an antibody against insulin and proinsulin (ab8403) purchased from Abcam (New Territories, Hong Kong). Specimens were observed using a visible-light microscope DP72 (Olympus, Tokyo, Japan). Pancreatic islet size was measured and calculated using Image-Pro Plus software v. 6.0 (Media Cybernetics, Washington, USA) with more than 100 islets randomly selected from five fields for every pancreatic slice from each experimental group. Specimens from healthy wild-type C57BL/J mice (WT) of the same age and genetic background were used for reference.

### Gene expression analysis

Total RNA was isolated from the liver and pancreas using Trizol reagent (Invitrogen, Shanghai, China) and an RNApure High-purity Total RNA Rapid Extraction Kit (spin column) (BioTeke, Beijing, China). RNA integrity and concentration were evaluated using a UVP GDS-7600 (California, USA) imaging instrument and a NanoDrop ND-1000 (Wilmington, USA) spectrophotometer, respectively. Roche NimbleGen Mouse Gene Expression 12×135 K arrays were used for gene expression microarray profiling analysis. All subsequent technical procedures and quality control analyses were performed at CapitalBio Corporation in Beijing, China [23]. Three independent replicates from each of the DMSe and DMCtrl groups were performed.

Differentially expressed genes (DEGs) were identified using the significance analysis of microarrays (SAM) method with SAMR software version 3.02 [24] with a selection threshold of the false discovery rate (q value (%)≤5). Differences in expression levels≥1.5 for up-regulation or<0.667 for down-regulation were considered statistically significant. Evaluation of DEGs according to Gene Ontology (GO) terms and Kyoto Encyclopedia of Genes and Genomes (KEGG) pathways were performed using the CapitalBio Molecule Annotation System (MAS) platform (http://bioinfo.capitalbio.com/mas).

### Proteomic analysis of liver samples

Liver samples were placed in liquid nitrogen and ground to a very fine powder using a mortar and pestle. The powder (approximately 100 mg per sample) was transferred to sterile tubes containing 1 ml ice-cold lysis buffer (30 mM Tris-HCl [pH 8.5], 7 M urea, 2 M thiourea, 4% CHAPS, and 20 µl/ml protease-inhibitor cocktail [Roche, Mannheim, Germany]) for protein extraction via ultrasonication (400 W for 5 min) in an ice bath. The homogenate was centrifuged at 4,000×*g* for 1 h at 4 °C, and the supernatant was collected to remove the fat layer and stored at −80 °C. A Micro BCA Protein Assay Kit (Thermo Scientific, Rockford, USA) was used to determine protein concentrations, and samples were adjusted to a final concentration of 5 mg/ml.

Three biological replicates from each of the two groups to be compared (50 µg of each protein sample) were labeled with Cy3 or Cy5 dye (GE Healthcare, Washington, USA). Additionally, a pooled sample was generated using equal amounts of all test samples and was labeled with Cy2 as an internal control. Labeled samples were analyzed using a Bio-Rad two-dimensional differential gel electrophoresis (2D-DIGE) system (Bio-Rad, Hercules, USA). Briefly, labeled samples were loaded onto 24-cm immobilized pH gradient strips (pH 3–10) and incubated at room temperature for 30–60 min. Isoelectric focusing was performed at 17 °C. The focused strips were equilibrated for 15 min in 6 M urea, 20% glycerol, 2% SDS, 375 mM Tris-HCl (pH 8.8), and 1% DTT, and then for an additional 15 min in the same buffer with DTT replaced by 4% iodoacetamide. After equilibration, proteins were separated on a 12% SDS polyacrylamide gel at 20 mA/gel overnight. The 2D gels were scanned using a Typhoon 9410 Scanner (GE Healthcare, Washington, USA), and imaging analysis was performed using DeCyder software version 5.02 (GE Healthcare, Washington, USA). Statistical significance was assessed using the Student *t* test; *P*-values≤0.05 were considered significant. Selected protein spots were cut out from the gels using a scalpel and digested with trypsin (Promega, Madison, WI) for protein peptide extraction. A MALDI-TOF 4800 mass spectrometer (Applied Biosystems, Foster City, CA) was used to identify differentially expressed proteins.

### Quantitative real-time polymerase chain reaction and western blot analyses

To validate the differential expression of genes and proteins detected using microarray analysis and 2D-DIGE, respectively, quantitative real-time polymerase chain reaction (qRT-PCR) assays and western blot analyses were performed. The cDNA was reverse-transcribed from total RNA using a First Strand cDNA Synthesis Kit (Fermentas, Beijing, China), and qRT-PCR assays were conducted using primers designed with NCBI Primer-BLAST (Table 1), a SYBR Green kit (Takara, Dalian, China), and an ABI-7500 Real-Time PCR system (Applied Biosystems, Foster City, CA). Gene expression levels were calculated according to the 2^−ΔΔCt^ method [25]. Antibodies against PAX6 (ab5790), NEUROD1 (ab60704), FBP2 (ab131253), PDX1 (ab47267), SCD1 (ab19862), GLUL (ab64613), and ALDOB (ab75751) were purchased from Abcam (New Territories, Hong Kong), and an antibody against GSTA1/2 (sc-323939) was purchased from Santa Cruz Biotechnology, Inc. (Santa Cruz, CA, USA). An anti-GAPDH antibody purchased from CST China (Shanghai, China) served as an internal reference. Protein samples were separated using SDS-PAGE and transferred to PVDF membranes. The blots were visualized using the SuperSignal West Pico Chemiluminescent Substrate (Thermo Scientific, Shanghai, China) and the intensity of protein bands was determined by densitometry using Gel-Pro Analyzer software v. 4.1 (Media Cybernetics, Washington, USA). The statistical significance of the differences between groups was estimated using the Student *t* test, with *P*<0.05 considered significant.

**Table 1:** Nucleotide sequence of primers used for qRT-PCR.

Gene Name	Forward (5′→3′)	Reverse (5′→3′)
*Pax6*	TTCCCGAATTCTGCAGACCC	TCTTGGCTTACTCCCTCCGA
*Neurod1*	CCCTACTCCTACCAGTCCCC	GAGGGGTCCGTCAAAGGAAG
*Fbp2*	CGCACCTTGGTCTATGGAGG	CCTCCTGCTTGCTCGATGAT
*Pdx1*	CCTTTCCCGAATGGAACCGA	TTCCGCTGTGTAAGCACCTC
*Scd1*	CACCTGCCTCTTCGGGATTT	TCTGAGAACTTGTGGTGGGC
*Pltp*	CGCAAAGGGCCACTTTTACTAC	GCCCCCATCATATAAGAACCAGT
*Aldob*	CCGCTTGCAGGAACAAACAA	ACGCCACTTCCCAAAGTCAA
*Glul*	TTATGGGAACAGACGGCCAC	TAACCTCCGCATTTGTCCCC
*Gsta1*	AGCCCGTGCTTCACTACTTC	CAATCTCCACCATGGGCACT
*InsR*	TCAAGACCAGACCCGAAGATTT	TCTCGAAGATAACCAGGGCATAG
*Gapdh*	CATGTTCCAGTATGACTCCACTC	GGCCTCACCCCATTTGATGT

## Results

### Insulin levels and hepatic oxidative stress capacity in diabetic mice treated with selenate

**Figure 1:**
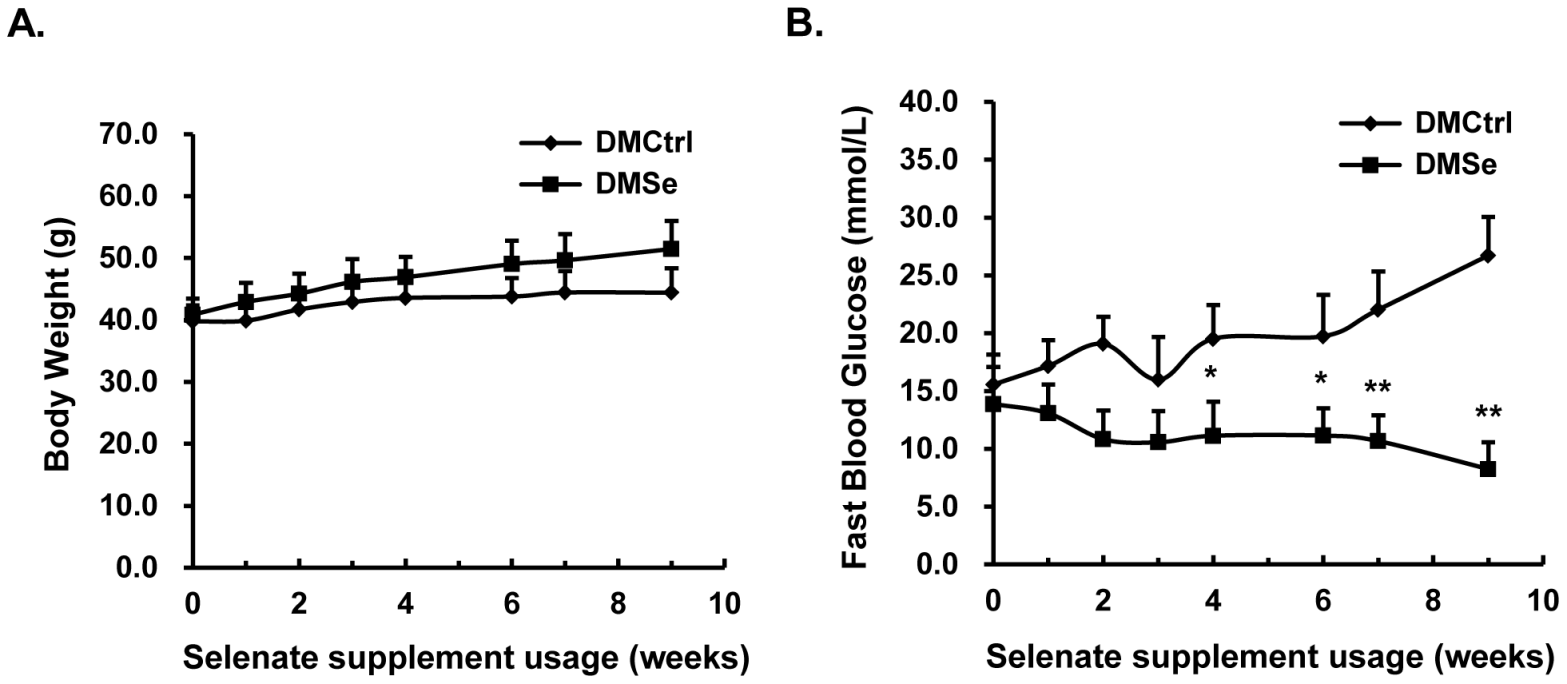
Phenotypic changes during selenate supplementation. (A) There were no differences in the changes in body weight between DMCtrl and DMSe mice. (B) Fasting blood glucose levels increased continuously in placebo-supplemented mice (DMCtrl), but decreased gradually in selenate-supplemented mice (DMSe) and returned to normal when supplementation ceased. Error bars were calculated for eight animals in the DMCtrl group and eight animals in the DMSe group. The asterisk (*) represents values found to be significantly different from the reference group (DMCtrl) using the Student *t* test (*P<0.005, **P<0.0001).

To investigate whether selenate supplementation affects animal health and the onset of diabetes, we monitored changes in food intake, weight gain, and fasting blood glucose levels in all experimental mice during 9 weeks of supplementation. All mice were in good condition, and there was no difference in the changes in BW between groups (Figure 1A). Fasting blood glucose levels increased continuously in DMCtrl mice, but decreased gradually in DMSe mice and had returned to near-normal levels by the end of the supplementation period (Figure 1B). In DMCtrl mice, the glycosylated hemoglobin content at time of sacrifice was markedly higher than during pretreatment, and this was accompanied by an increase in glucose level. In contrast, the level of glycosylated hemoglobin in DMSe mice did not change. The plasma insulin concentration of DMSe mice was nearly twice that of controls, and there was a 30% increase in insulin protein levels in pancreatic tissue homogenate. Compared with the control group, the DMSe mice had increased total plasma cholesterol (132%) and low-density lipoprotein (116%) levels, but decreased levels of plasma TG (51.7%) and MDA (73.3%, *P* = 0.06) (Table 2).

**Table 2:** Biochemical parameters (Mean±SD) #of the *db/db* mice with or without dietary selenate supplementation.

Parameter	DMCtrl	DMSe	*P* Value
**Blood**			
Pretreatment: HbA1c [%(mmol/mol)]	4.2±0.3(21±2)	4.0±0.5(20±6)	
At sacrifice: HbA1c [%(mmol/mol)]	5.9±0.6(41±6)	4.4±0.5(25±5)**	0.00516 (0.00331)
**Plasma**			
glucose, mmol/L	29.26±6.52	15.99±5.21***	0.00097
Insulin, pmol/L	11.78±1.67	21.44±8.16*	0.01640
HOMA-IR index	16.56±3.12	13.56±1.91^NS^	0.09395
triglycerides, mmol/L	1.18±0.39	0.61±0.22*	0.04155
cholesterol, mmol/L	2.74±0.22	3.61±0.60*	0.04061
low-density lipoprotein, mmol/L	1.91±0.08	2.21±0.23*	0.01206
malondialdehyde, mmol/L	3.71±0.73	2.72±0.73^NS^	0.06013
**Pancreatic tissue homogenate**			
Insulin, pmol/mg protein	282.20±57.24	366.25±58.48*	0.01668

# Mean±SD were calculated for five animals in the DMCtrl group and eight animals in the DMSe group.

*Values significantly different from DMCtrl mice, by the Student *t* test (*P<0.05, **P<0.01, *** *P*<0.001).

NS indicates the values were not significantly different from DMCtrl mice by the Student *t* test (*P*>0.05).

The activity of antioxidant enzymes such as GSH-Px, CAT and SOD were measured to estimate the oxidative stress capacity of *db/db* mice after selenate supplementation. There was a slight decrease in hepatic GSH-Px activity, but a small increase in hepatic SOD activity. However, no significant difference in hepatic CAT activity was found between the two groups, and the global levels of GSH-Px activity, SOD activity and GSH content were also unchanged (Table 3).

**Table 3:** Oxidative stress parameters (Mean±SD) #of the *db/db* mice with or without dietary selenate supplementation.

Parameter	DMCtrl	DMSe	*P* Value
**Plasma**			
GSH, µmol/L	31.42±8.67	33.85±9.93	NS
GSH-Px activity, U/ml	85.54±11.66	79.81±13.06	NS
SOD activity, U/ml	124.38±41.67	133.76±31.54	NS
**Hepatic**			
glycogen, mg/g tissue	31.79±8.10	26.26±7.91	NS
CAT activity, U/mg protein	10.93±2.54	12.515±2.10	NS
SOD activity, U/mg protein	38.37±10.74	55.35±10.99**	0.00712
GSH-Px activity, U/mg protein	1183.54±98.74	1037.93±117.18*	0.02508

# Mean±SD were calculated for eight animals in the DMCtrl group and eight animals in the DMSe group.

*Values significantly different from DMCtrl mice, by the Student *t* test (*P<0.05, **P<0.01).

NS means no significance (*P*>0.05) between the DMCtrl group and DMSe group.

### Increase in pancreatic islet size and accumulation of liver fat in mice receiving selenate supplementation

**Figure 2:**
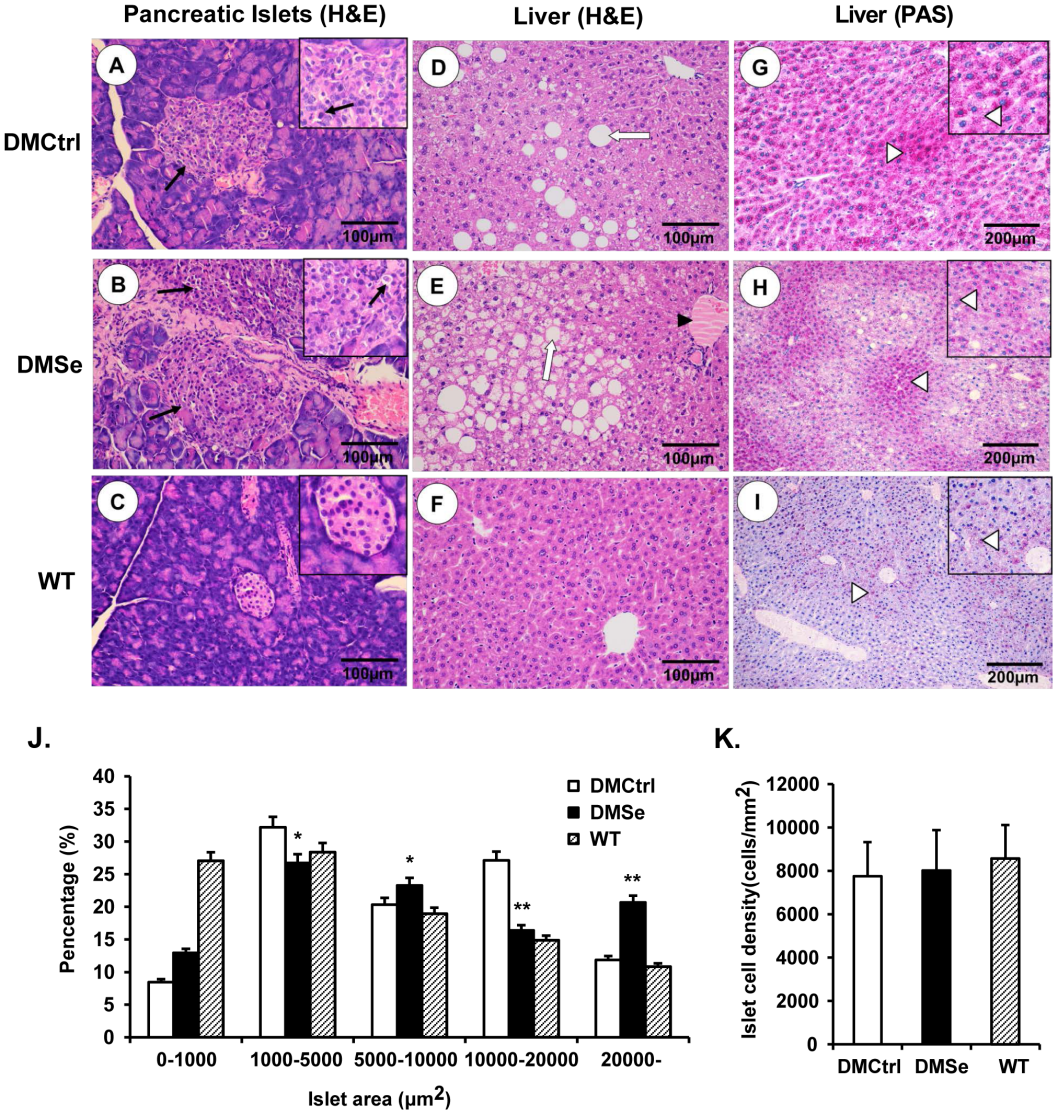
Histological analysis of pathological changes in the pancreas and liver. Compared with the results from the DMCtrl group (A), the pancreatic islets of selenate-treated DMSe mice (B) increased in size. Occasional capillary congestion and islet cell necrosis (black arrows) was observed. (C) Pancreatic islets of WT mice were shaped regularly and arranged evenly. (D) Modest fatty liver degeneration and vacuolization was observed in the DMCtrl control group, but (E) this was exacerbated after selenate treatment and was accompanied by a large number of swollen hepatocytes and lipid vacuoles (white arrows). Steatosis and hepatic cord congestion (black triangles) were also present. (F) There were no abnormal lesions in the liver tissue of WT mice. (H) PAS staining for hepatic glycogen (purple-reddish granules, white triangles) revealed a reduced capacity for glycogen storage, along with increased fatty liver damage in DMSe mice compared with (G) that in DMCtrl mice. (I) There were occasional purple-reddish glycogen granules in the liver tissue of WT mice. Changes in islet area (J) and islet cell density (K) were measured and calculated using Image-Pro Plus software v. 6.0 (Media Cybernetics, Washington, USA) from more than 100 randomly selected islets in five fields from each pancreatic slice from each experimental group. The asterisk (*) represents significant differences (*P<0.05, **P<0.01) between the DMSe group and DMCtrl group.

H&E staining was performed in order to analyze pathological changes in the pancreas and liver histologically, while PAS staining was used to determine hepatic glycogen storage capacity (Figure 2). Compared to WT mice, where the area of the islets was mostly less than 10000 µm^2^ (approximately 74.33%), we observed a dramatic increase in pancreatic islet size in DMCtrl mice, especially the percentage between 10000 µm^2^ and 20000 µm^2^ in area, which increased from 14.86% to 27.12%. A further increase in islet area occurred in DMSe mice after selenate supplementation, with 20.69% of the islets greater than 20000 µm^2^ (Figure 2J). Although the islets were irregularly shaped and loosely arranged in diabetic *db/db* mice, there was no significant difference in islet cell density between WT mice and *db/db* mice with or without selenate treatment (Figure 2K). Occasional capillary congestion and islet cell necrosis was observed in both DMCtrl and DMSe mice.

Limited instances of fatty degeneration and vacuolization were observed in the livers of DMCtrl mice; however, selenate treatment exacerbated these lesions, which featured large numbers of swollen hepatocytes, lipid vacuoles, steatosis, and hepatic cord congestion. Moreover, hepatic glycogen storage capacity was reduced in DMSe mice.

**Figure 3:**
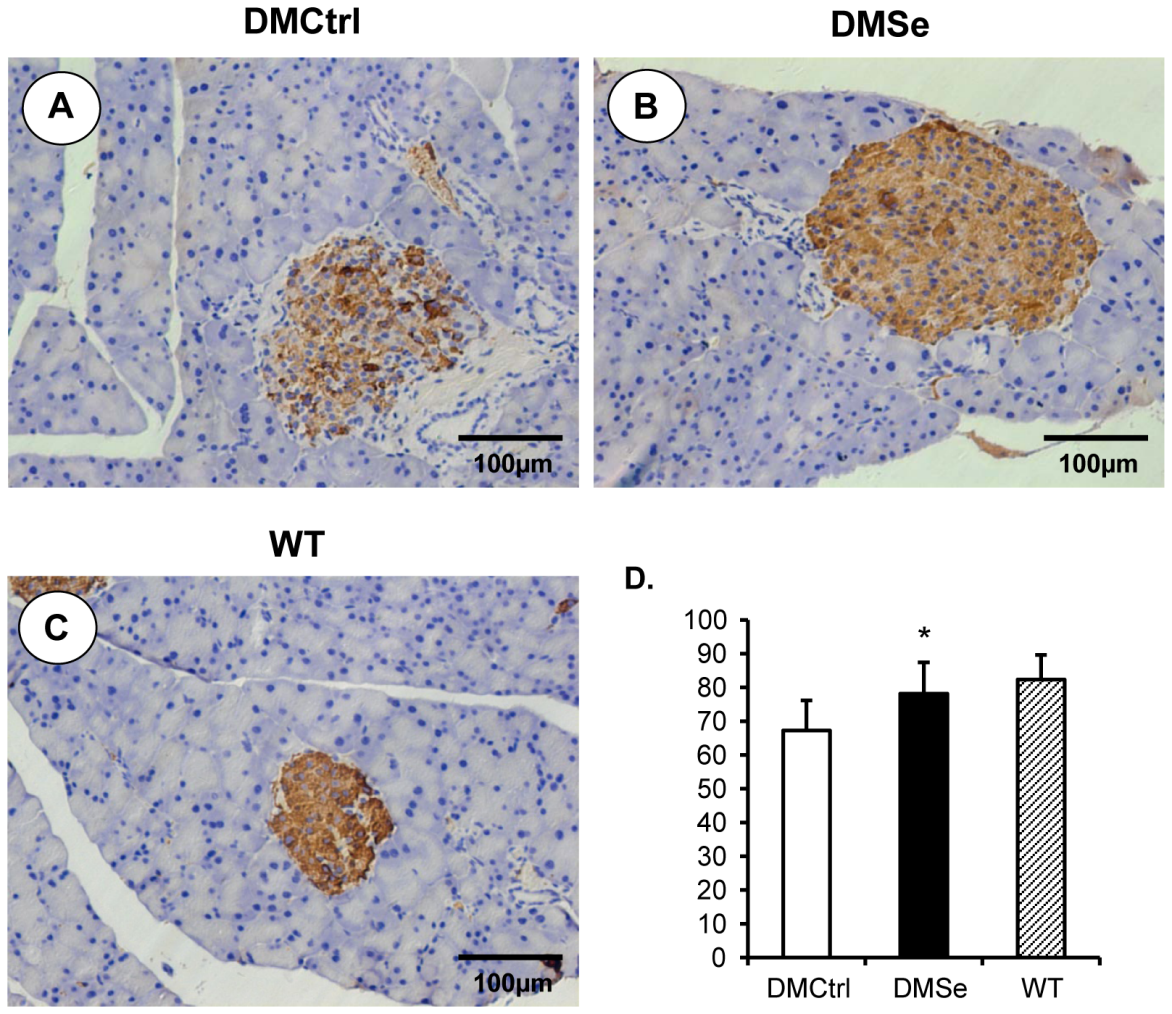
Immunohistochemical analysis to measure the production of insulin. Positive cells displayed brownish yellow granules in the cytoplasm. (A) Although islet size was increased in DMCtrl mice, the relative positive rate of insulin expression was decreased as compared with WT mice (C). (B) Selenate treatment relieved the decline and maintained a similar levels of insulin expression in both DMSe mice and WT mice. (D) The insulin positive ratios of cells were measured and calculated using Image-Pro Plus software v. 6.0 (Media Cybernetics, Washington, USA) from more than 100 islets randomly selected in five fields for every pancreatic slice of each experimental group. The asterisk (*) represents significant differences (*P<0.01) between the DMSe group and DMCtrl group.

In addition, immunohistochemical analysis was performed to measure the synthesis and secretion of insulin. Although islet size was increased in DMCtrl mice compared with WT mice, the relative positive rate of insulin expression decreased by 15.14%. Selenate treatment relieved the effect, resulting in a decline of only 4.22% in DMSe mice compared to WT mice (Figure 3).

### Analysis of expression levels of genes associated with pancreatic β-cell function

**Figure 4:**
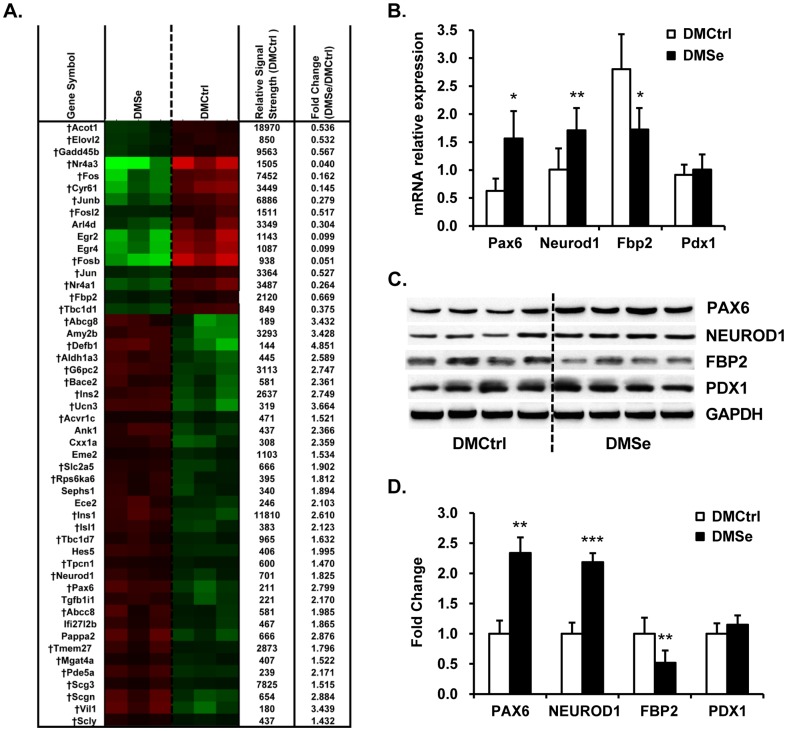
Pancreatic-specific genes and proteins differentially expressed in *db/db* mice. (A) Heat map showing differences in the mRNA expression levels (green, lower; red, higher between DMCtrl and DMSe groups). The (†) indicates genes that are closely linked to key pancreatic β-cell functions or the development of diabetes. The relative signal strength represents the mean gene expression value in the DMCtrl group derived from Robust Multi-Array analysis. (B) qRT-PCR analysis of *Pax6*, *Neurod1*, *Fbp2*, and *Pdx1* expression was used to validate the cDNA microarray data from the pancreas. There was strong consistency between the two assays. Three replicates were performed for each mouse (eight mice per group). *Gapdh* was used as an internal reference gene. The results are presented as the relative changes in mRNA expression levels in DMSe vs. DMCtrl mice. (C) Representative western blots and (D) quantitation of the levels of PAX6, NEUROD1, FBP2 and PDX1. GAPDH was used as an internal reference. Protein band intensity was quantified using densitometry using Gel-Pro Analyzer software v. 4.1 (Media Cybernetics, Washington, USA), and the results are presented as the ratio of densitometric values from DMSe to DMCtrl. The asterisk (*) represents values shown to be significantly different from DMCtrl by the Student *t* test (*P<0.05, **P<0.01, ***P<0.001).

Among the differentially expressed genes in the pancreas, 81 were upregulated and 34 were downregulated in DMSe mice (Table S1). A wide range of genes were closely associated with either key pancreatic β-cell functions or the development of diabetes (Figure 4A). Of these, there were increases in the levels of mRNAs encoding transcription factors that regulate insulin synthesis and secretion, such as paired box gene 6 (*Pax6*), neurogenic differentiation 1 (*Neurod1*), and activin A receptor type 1c (*Acvr1c*). We detected elevated levels of mRNAs encoding components of the insulin signal transduction pathway, such as insulin (*Ins1* and *Ins2*), ribosomal protein S6 kinase polypeptide 6 (*Rps6ka6*), and doublecortin-like kinase 2 (*Dclk2*). In contrast, the level of *c-Jun* expression decreased. The transcription of genes encoding proteins involved in glucolipid metabolism, including glucose-6-phosphatase catalytic subunit 2 (*G6pc2*), transforming growth factor beta 1 induced transcript 1 (*Tgfb1i1*), solute carrier family 2 member 5 (*Slc2a5*), ATP-binding cassette sub-family G member 8 (*Abcg8*), and ATP-binding cassette sub-family C member 8 (*Abcc8*), was induced. However, the levels of fructose bisphosphatase 2 (*Fbp2*), elongation of very long chain fatty acids (*Elovl2*), and acyl-CoA thioesterase 1 (*Acot1*) were suppressed.

Along with the increase in pancreatic islet size, as determined by histological analysis in *db/db* mice treated with selenate (DMSe), the expression of cell-cycle components was also elevated. Transcription of genes encoding proteins known to stimulate cell proliferation, such as *Spc25*, *Cdc27* and *Rprm*, increased while the transcription of genes that encode proteins that inhibit cell cycle progression, such as *Jun*, *Junb*, and *Fosl2*, was decreased. The expression levels of apoptosis-associated genes, including growth arrest and DNA-damage-inducible 45 beta (*Gadd45b*), nuclear receptor (*Nr4a1*), and B cell translocation gene 2, anti-proliferative (*Btg2*), decreased. Moreover, transcription levels of genes encoding proteins that mediate selenide metabolism, such as selenophosphate synthetase 1 (*Sephs1*) and selenocysteine lyase (*Scly*), also increased, whereas the expression of genes encoding major selenoproteins, such as glutathione peroxidase 1 (*GPx1*), selenoprotein P (*Sepp1*), and 15 kD selenoprotein (*15-Sep*), did not change significantly. These findings indicate that supplementation with dietary selenate did not affect the overall expression of selenoproteins in the transcriptome.

Differential expression of the genes of interest (*Pax6*, *Neurod1*, *Fbp2*, and *Pdx1*) and their encoded proteins (PAX6, NEUROD1, FBP2 and PDX1) was validated using qRT-PCR (Figure 4B) and western blot analyses (Figure 4C, 4D), and the results showed coordinated changes in mRNA and protein levels.

### Analysis of the expression of genes involved in lipogenesis and inflammation

**Figure 5:**
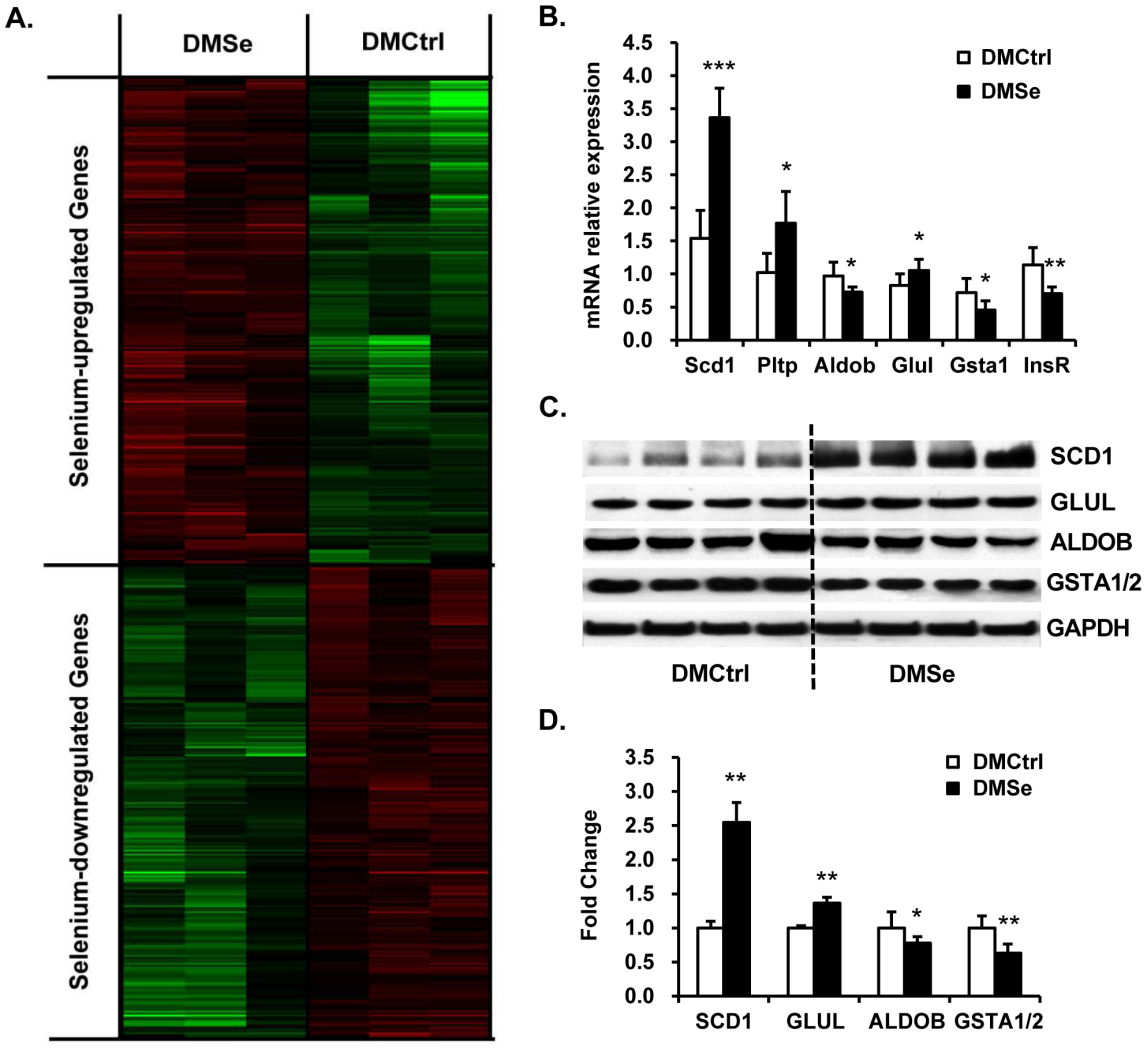
Hepatic genes and proteins differentially expressed between DMCtrl and DMSe groups. (A) The heat map depicts differences in mRNA expression levels according to brightness (green, downregulated; red, upregulated compared with DMCtrl mice). (B) qRT-PCR analysis of *Scd1*, *Pltp*, *Aldob*, *Glul*, *Gsta1* and *InsR*, which were selected to validate the cDNA microarray data from the liver. The data indicate excellent consistency between the two techniques. Three replicates were performed for each mouse (eight mice per group). *Gapdh* was used as an internal reference gene. The results are presented as the relative changes in mRNA expression levels in DMSe vs. DMCtrl mice. (C) Representative western blots and (D) quantitation of selected proteins. There was agreement in the expression levels of GLUL and ALDOB as detected using 2D-DIGE and western blot analyses. SCD1 and GSTA1/2 displayed the same differences in expression level seen in the microarray data. GAPDH was used as an internal reference. The intensity of protein bands was quantified by densitometry using Gel-Pro Analyzer software v. 4.1 (Media Cybernetics, Washington, USA), and the results are presented as the ratio of densitometric values from DMSe to DMCtrl. The asterisk (*) represents values shown to be significantly different from DMCtrl by the Student *t* test (*P<0.05, **P<0.01, ***P<0.001).

DNA microarray analysis of liver samples showed that the expression levels of 582 transcripts had changed by a factor of≥1.5 in response to selenate supplementation (Table S2). The levels of 301 genes were elevated while 281 genes were decreased (Figure 5A). Functional annotation and pathway analysis showed that a large proportion of these differentially expressed genes were involved in regulating lipid metabolism, including genes involved in fatty acid synthesis through arachidonic acid metabolism and the PPARα signaling pathway. The other differentially expressed genes were mainly associated with detoxification, inflammatory response, and cell cycle regulation.

We found that genes encoding key enzymes in fatty acid synthesis, such as prostaglandin D2 synthase (*Ptgds*), stearoyl-coenzyme A desaturase 1 (*Scd1*), acetyl-coenzyme A carboxylase beta (*Acacb*), NADP-dependent malic enzyme 1 (*Me1*), and elongation of very long chain fatty acids (*Elovl5*), were upregulated in the DMSe mice. Conversely, the gene encoding acyl-CoA synthetase medium-chain family member 5 (*Acsm5*) was downregulated in these animals. Carnitine palmitoyltransferase 1b (*Cpt1b*), hydroxyacid oxidase 2 (*Hao2*), and adiponectin receptor 2 (*Adipor2*), are involved in fatty acid oxidation and were induced by selenate supplementation. The transcription of phospholipid transfer protein (*Pltp*) and 3-hydroxy-3-methylglutaryl-Coenzyme A synthase 1 (*Hmgcs1*), which function in ketogenesis, was increased in response to selenate treatment. Moreover, the transcription of genes ncoding multiple cytochrome P450 isoforms, including *Cyp2a4*, *Cyp2b10*, *Cyp2c44*, *Cyp4a12b*, and *Cyp7b1*, was significantly downregulated in response to selenate treatment. The expression of other genes, such as hydroxysteroid 11-beta dehydrogenase 1 (*Hsd11b1*), lysosomal acid lipase A (*Lipa*), and phospholipase (*Plcg1*), was also downregulated. Most of the differentially expressed genes listed above encode components of the PPAR-α signaling pathway that function to induce the synthesis of fatty acids and ketone bodies in the liver.

Genes that encode proteins involved in detoxification or the response to oxidative stress, including serine (or cysteine) peptidase inhibitor subfamily members (*SerpinA1a*, *SerpinA1b*, *SerpinA1d*, *SerpinA3f*, *SerpinA3k* and *SerpinA3m*), were expressed at lower levels in the DMSe group than that in the DMCtrl group. Expression of the glutathione *S*-transferase (GST) family genes *Gsta1* and *Gsta2* was downregulated in the DMSe group, while *Gstm3* expression was upregulated.

Consistent with our histological data indicating exacerbated inflammation and necrosis in DMSe mice, our expression profiling data indicated the activation of inflammatory stress pathways. In the DMSe group, we detected increased levels of expression of genes encoding chemokines involved in the inflammatory response (*Flt1* and *Ccl19*), whereas expression of *Cxcl1* and *Cx3cl1* decreased. Similarly, the expression levels of certain apoptosis-related genes were significantly altered by selenate treatment. There was increased expression of genes encoding the apoptosis-inducing factors *Aifm1* and *Aifm3*, cell death-inducing DNA fragmentation factor alpha subunit-like effector A and C (*Cidea*, *Cidec*), death associated protein-like 1 (*Dapl1*), MAP-kinase activating death domain (*Madd*), DNA damage-inducible transcript 4 (*Ddit4*), and gap junction protein beta 6 (*Gjb6*).

All of the data as well as detailed information from the microarray analysis were deposited in the NCBI-GEO database, with the accession number GSE55636.

Proteomic analysis of liver using 2D-DIGE and MALDI-TOF/MS-MS identified five proteins whose expression were significantly changed in DMSe mice compared with DMCtrl mice, although the differences were modest (Table 4). There was no direct correlation between the results of the cDNA microarray analysis and the proteomic analysis, with the exception of aldolase B fructose-bisphosphate (ALDOB). Analysis of these proteins using the online biological software DAVID (http://david.abcc.ncifcrf.gov/) revealed that they represented two functions: (i) proteins involved in glucolipid metabolism, such as aldolase B (ALDOB), glutamate-ammonia ligase (GLUL), and isocitrate dehydrogenase 1 (NADP+) soluble (IDH1); and (ii) proteins involved in the endoplasmic reticulum assembly pathway, such as protein disulfide isomerase associated 3 (PDIA3) and Calreticulin (CALR).

**Table 4:** Liver proteins differentially expressed in *db/db* mice with or without dietary selenate supplementation.

Protein name	Accession number#	Symbols	Fold change DMSe vs. DMCtrl	*P.* Value
Protein disulfide-isomerase A3 precursor	gi|112293264	Pdia3	1.36	0.0410
Glutamate-ammonia ligase (glutamine synthetase)	gi|31982332	Glul	1.27	0.0035
Calreticulin precursor	gi|6680836	Calr	1.25	0.0400
Isocitrate dehydrogenase 1 (NADP^+^), soluble	gi|57242927	Idh1	1.22	0.0330
Aldolase B, fructose-bisphosphate	gi|21707669	Aldob	−1.35	0.0490

# NCBI database accession number.

The differential expression of genes such as *Scd1*, *Pltp*, *Aldob*, *Glul*, *Gsta1* and *InsR* was validated by qRT-PCR. The results of cDNA microarray and qRT-PCR analyses were essentially consistent (Figure 5B). The results of western blot analysis for representative proteins such as GLUL, ALDOB, SCD1 and GSTA1/2 showed changes in protein levels between the two treatment groups (Figure 5C, D), and these differences were similar to those found by cDNA microarray analysis and 2D-DIGE coupled with mass spectrometry analysis.

## Discussion

*In vivo* and *in vitro* studies have demonstrated the insulin-mimetic properties of selenium and have indicated that appropriate dietary supplementation with selenium can prevent diabetes. However, different forms of selenium have differing effects on insulin-regulated carbohydrate metabolism, indicating various functional mechanisms of selenium compounds. For example, selenate may suppress the increase in fasting plasma glucose concentrations in diabetes models by increasing insulin sensitivity and by acting as an insulin-mimetic in liver and adipose tissue [11], [20],[26], while selenite has been found to stimulate insulin production and secretion from islets and further enhance carbohydrate efficiency via high insulin levels [27]. It has even been reported that selenite may counteract insulin-induced signaling [28]. In the present study, after oral selenate administration for 9 weeks the increase in fasting blood glucose in diabetic *db/db* mice was suppressed, while plasma insulin concentrations increased significantly. The pancreatic islets were enlarged and the expression of insulin protein in the pancreas was also upregulated. Additionally, the insulin transcription factors Pax6 and Neurod1, which are important for the regulation of insulin synthesis and secretion [29], [30], were also upregulated at the mRNA and protein expression levels. Thus, dietary selenate supplementation has a similar effect on enhancing islet function. Although a previous report [27] indicated that selenate stimulates the *Ipf1* (insulin promoter factor 1) gene promoter to a lesser extent than selenite, the authors concluded that this was due to the presence of specific selenate reductase activity in the pancreatic extract. This blocked the reduction of selenate to selenite, which is readily reduced to selenide and subsequently assimilated into selenoproteins. Another study found that free SeIV compounds, the cellular metabolic intermediates of selenate, act as inhibitors of protein tyrosine phosphatases (PTPs), which is why increasing selenate concentration directly *in vitro* effected no inhibition of PTPs [20]. Taking this into consideration, we propose that the mechanism by which selenate stimulates insulin production may also be associated with a metabolic intermediate product of selenate. Unfortunately, our results failed to prove this hypothesis, which remains to be confirmed by future research.

Unexpectedly, hepatopathy observed in the DMSe mice was more severe than that in the DMCtrl mice and was accompanied by increased hepatocyte lipid vacuolation and hepatic cord congestion. Levels of plasma cholesterol and low-density lipoprotein were increased while triglyceride levels decreased, and cDNA microarray analysis detected upregulation of lipogenic genes, which may account for this fat accumulation [31]. Of particular interest are genes involved in fatty acid oxidation, which could cause an increase in reactive oxygen species (ROS) and oxidative stress [32]. The HOMA-IR index, calculated based on the levels of fasting plasma glucose and fasting plasma insulin, declined in DMSe mice compared with DMCtrl mice, although the effect was not significant. However, there was no difference in expression of genes or proteins involved in the insulin signaling cascade, including PTP1B, which can be suppressed by selenate metabolic intermediates [20].

We observed that the expression of a group of genes that encode proteins with oxidoreductase activity in the liver was suppressed. These included cytochrome P450 family genes related to arachidonic acid metabolism [33], which generate metabolites such as eicosatetraenoic acids that activate the MAPK and PI3K/AKT signaling pathways [34], enhance insulin sensitivity, and inhibit hepatic inflammation [35] as well as apoptosis [36]. Further, several enzymes of the GST family known to be involved in ROS detoxification [37] were down-regulated, indicating a possible reduction in antioxidant defense capability. Moreover, there was no difference in the expression of any selenoproteins between the DMSe and DMCtrl groups, although hepatic GSH-Px activity decreased. Thus, we conclude that oral selenate administration did not increase insulin sensitivity, but instead reduced antioxidant defense capacity and exacerbated fatty liver degeneration in *db/db* mice, in contrast to what has been reported in previous studies [26], [38].

The proteomic data presented here provide novel insight on the effects of selenate supplementation in diabetic *db/db* mice. Aldolase B (ALDOB) is an important enzyme in glycolysis and gluconeogenesis that catalyzes the dissimilation of fructose 1, 6-bisphosphate (FBP) or fructose 1-phosphate (F1P) [39]. Mutations leading to defects in aldolase B result in a condition known as hereditary fructose intolerance [40], where the lack of functional aldolase B leads to the accumulation of F1P in bodily tissues. This accumulation damages tissues and traps phosphate in an unusable form that does not return to the general phosphate pool, eventually depleting phosphate and ATP stores [41]. The lack of readily available phosphate terminates glycogenolysis in the liver, which causes hypoglycemia [41]. The accumulation of F1P also inhibits glycogenolysis and further reduces blood glucose levels [41]. ALDOB was downregulated in DMSe mice in this study, indicating decreased functional aldolase B levels that may account for the reduction in blood glucose levels.

Our liver proteome analysis also found that, hepatic glutamine ligase (GLUL) and NADP+-dependent isocitrate dehydrogenase 1 (IDH1) were upregulated in DMSe mice. GLUL (glutamine synthetase, GS) catalyzes the ATP-dependent reaction of glutamate with ammonia that yields glutamine, and also plays an important role in the regulation of nitrogen metabolism [42], [43]. Recent studies have found that selenium affects nitrogen and glutamate metabolism via regulation of GS expression in lettuce plants [43], although the specific mechanism involved is unknown. Isocitrate dehydrogenases catalyze the oxidative decarboxylation of isocitrate to 2-oxoglutarate, generating NADPH. IDH1 plays a vital role in the regulation of lipogenesis and metabolism, and the phenotypes of transgenic mice that overexpress IDH1 include fatty liver, hyperlipidemia, and obesity [44], [45]. Protein disulfide isomerase associated 3 (PDIA3) and Calreticulin (CALR) were also upregulated after selenate treatment in our study. PDIA3, also known as ERp57, is a thiol oxidoreductase with protein disulfide isomerase activity and is a rate-limiting enzyme for protein folding in the endoplasmic reticulum [46]. Calreticulin, an endoplasmic reticulum chaperone and calcium regulator, plays an important role in regulating collagen expression and fibrosis [47]. PDIA3 and CALR act together in the major histocompatibility complex class I assembly pathway [46], [48]. The increased expression of these two endoplasmic reticulum proteins in DMSe mice indicate increased endoplasmic reticulum stress and the progression of hepatic steatosis to fibrosis.

Based on the above-mentioned phenotypes, it is possible that the doses applied in the present study resulted in chronic selenium toxicity. A standard mouse chow diet made with sodium selenite contains 0.2 mg Se/kg, and our supplementation dose was 0.8 mg sodium selenate/kg body weight, which would correspond to approximately 0.336 mg Se/kg body weight. We assume the consumption of diet per day is one-tenth of the body weight and that a 50 g mouse would consume 5 g diet per day. Thus, the supplemented amount is approximately 16.8-fold the recommended dietary amount (0.2 mg Se/kg diet). It has been previously reported that [20] selenium concentrations up to 20-fold over the recommended amount (4 mgSe/kg diet) do not affect animal health or mortality after 2 years (survival rate > 90%). However, after careful consideration of the phenotypes mentioned in this manuscript, we concluded that chronic selenium toxicity was a distinct possibility, especially in liver tissue, which is the most sensitive organ to selenium toxicity.

In conclusion, our findings suggest that nutritional supplementation of *db/db* mice with selenate does not improve or prevent the symptoms of type 2 diabetes, although hyperglycemia was decreased as a result of increasing insulin production and secretion. Moreover, long-term hyperinsulinemia eventually led to reduced antioxidant defense capacity and exacerbated fatty liver degeneration.

## Supporting Information

Table S1 Expression information of all the differential genes with a change factor of ≥ 1.5 in pancreas.
(XLS)

Table S2 Expression information of all the differential genes with a change factor of ≥ 1.5 in liver.
(XLS)
